# Different ascending aortic phenotypes with similar mutations in 2 patients with Loeys-Dietz syndrome type 2

**DOI:** 10.1093/icvts/ivac159

**Published:** 2022-06-11

**Authors:** Roland Heck, Björn Fischer-Zirnsak, Joachim Photiadis, Denise Horn, Petra Gehle

**Affiliations:** Department of Cardiothoracic and Vascular Surgery, German Heart Center Berlin, Berlin, Germany; Department of Medical Genetics and Human Genetics, Charité – Universitätsmedizin Berlin, corporate member of Freie Universität Berlin, Humboldt Universität zu Berlin, and Berlin Institute of Health, Berlin, Germany; Max Planck Institute for Molecular Genetics FG Development and Disease, Berlin, Germany; Department of Congenital Heart Surgery, Pediatric Heart Surgery, German Heart Center Berlin, Berlin, Germany; Department of Medical Genetics and Human Genetics, Charité – Universitätsmedizin Berlin, corporate member of Freie Universität Berlin, Humboldt Universität zu Berlin, and Berlin Institute of Health, Berlin, Germany; Department of Cardiology, Charité – Universitätsmedizin Berlin, Berlin, Germany

**Keywords:** Great vessel anomaly, Aortic Surgery, Loeys-Dietz syndrome

## Abstract

Our goal was to present 2 infants with confirmed Loeys-Dietz syndrome. The missense mutations in exon 7 of the *TGFBR2* gene are only 5 codons apart (c.1597T>C and c.1582C>G). Phenotypically, the aneurysms of the ascending aorta were restricted to different segments of the aorta: the suprajunctional segment in 1 patient and the aortic root in another. These cases highlight the complexity of signaling pathways and gene expression in the pathogenesis of aortic aneurysms.

## INTRODUCTION

**Figure 1:**
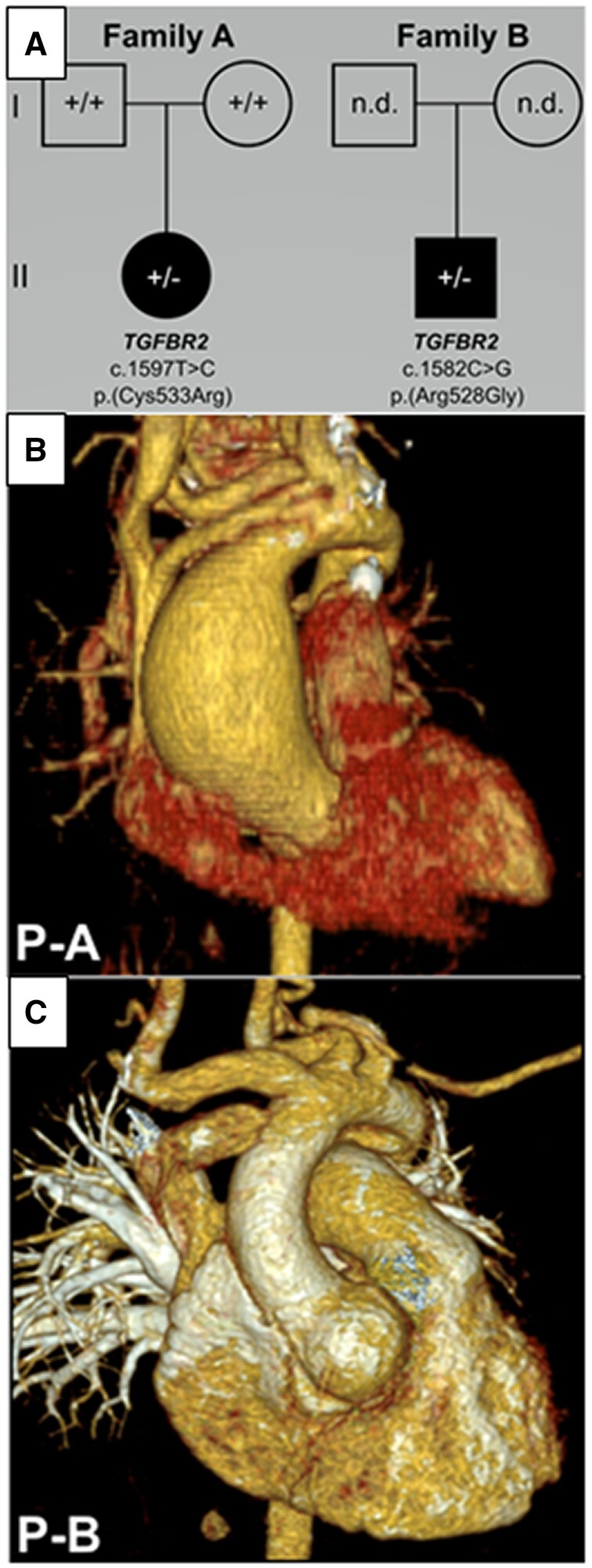
(**A**) Family tree of the 2 patients. (**B**) Preoperative 3-dimensional computed tomography of the suprajunctional ascending aortic aneurysm of patient A. (**C**) Preoperative 3-dimensional computed tomography of the aortic root aneurysm of patient B.

Loeys-Dietz syndrome (LDS) is an autosomal-dominant connective tissue disorder due to pathogenic variants that affect the transforming growth factor ß receptors I and II, SMAD 2 and 3 and the transforming growth factors ß I and II. Its clinical manifestations include arterial aneurysms and tortuosity with skeletal manifestations such as cleft palate or pectus excavatum. Intrafamilial variations in phenotypes and their severity are common [1]. The independent factor for increased mortality is an aortic dissection that can lead to sudden aortic rupture [2]. Frequent monitoring of aneurysm progression and adequate timing of surgery to prevent adverse outcomes are mandatory. Valve-sparing aortic root surgery is the treatment of choice in the young affected cohort [3]. In this case series, we present 2 infants with genetically confirmed Loeys-Dietz syndrome (OMIM# LDS2 610168) due to pathogenic variants in *TGFBR2* on chromosome 3p24.1, encoding the transforming growth factor ß receptor 2. The identified missense variants affect the TGFBR2 polypeptide at positions p.(Arg528Gly) and p.(Cys533Arg), respectively, lying only 5 codons apart. Phenotypically, the aneurysms of the ascending aorta were restricted to different segments of the aorta: the suprajunctional segment in patient A and the aortic root in patient B (Fig. 1). These cases highlight the complexity of the phenotype due to similar genetic alterations. The ethics committee of the Charité – Universitätsmedizin Berlin approved this investigation (application number: EA2/120/16).

## PATIENT A

The first presentation of the female patient in our aortic outpatient department at the age of 22 months was due to a suspected connective tissue disorder. She already had surgically fixed club feet, unstable pes/talipes calcaneus, pectus excavatum (sunken chest), high-arched palate, uvula bifida, tooth malposition, arachnodactyly, scoliosis, positive wrist and thumb sign, joint contraction elbow, hypertelorism, amblyopia, anisometropia and psychomotor delay (physical and speech development disorders). A missense variant in exon 7 of *TGFBR2* (NM_003242.6: c.1597T>C (p.Cys533Arg) was identified using Sanger sequencing. Cysteine was exchanged with arginine at position 533; this variant is listed in the Human Gene Mutation Database (CM064332) as a disease mutation. Co-segregation analyses revealed this variant to be absent in the parents; thus we considered it to be a *de novo* alteration. A highly conserved kinase domain was affected, so the function of the receptor should be modified. The aortic diameters were assessed frequently. From 25 mm (*z*-score: +5.16) at the time of her first presentation, the diameter of her ascending aorta increased to 39 mm (*z*-score: +10.1) over a 24 -month period [4]. Additionally, echocardiography showed aortic regurgitation grade II°, a dilated pulmonary trunk and a multisegment mitral valve prolapse with mild regurgitation (grade I°) (Table 1).

**Table 1:** Clinical and imaging parameters of the 2 observed patients

	Patient A	Patient B
Demographics		
Age, years	4	5
Sex	Female	Male
Height, cm	103	107
Weight, kg	16.4	15.6
Echocardiography		
Aortic regurgitation	2°	1°
Mitral regurgitation	1°	1°
Mitral valve prolapse	Yes	Yes
Left ventricular ejection fraction	57%	55%
Left ventricular end-diastolic diameter	38	44
Bicuspid aortic valve	Yes	No
Aortic dimensions		
Aortic annulus	20	23
Bulbus aortae	30	46
Sinotubular junction	38	22
Ascending aortae	39	19
*Z*-scores		
Aortic annulus	4.06	5.33
Bulbus aortae	5.35	9.48
Sinotubular junction	8.94	3.88
Ascending aortae	10.11	2.14
Congenital heart defects		
Persistent foramen ovale	Yes	Yes
Persistent ductus arteriosus	Yes	Yes
Vascular disorders		
Arterial tortuosity of arch vessels	Yes	Yes
Arterial tortuosity of cranial vessels	No	No
Skeletal disorders		
Pectus excavatum	Yes	Yes
Upper-to-lower segment ratio < 0.85 or arm span-to-height ratio > 1.05	Yes	Yes
Positive wrist and thumb sign	Yes	Yes
Pes planus	Yes	Yes
Scoliosis >20°	Yes	Yes
Extension at elbows <170°	Yes	No
Dural ectasia	Yes	Yes
Facial appearance		
Craniosynostosis	Yes	Yes
Hypertelorism	Yes	No
High-arched palate	Yes	Yes
Uvula bifida/cleft palate	Yes	Yes
Tooth malposition	Yes	No
Arachnodactyly	Yes	Yes
Ocular manifestations		
Amblyopia	Yes	Yes
Anisometropia	Yes	Yes
Physical and speech development disorders	Yes	Yes
Exotropia, exophoria	Yes	Yes

## PATIENT B

The first presentation of the male patient in our aortic outpatient department at the age of 36 months was due to typical skeletal anomalies that led to the identification of a suspected connective tissue disorder. At the physical examination, pectus excavatum, craniosynostosis, Chiari I malformation with low tonsillar and medulla compression, cleft palate, pes valgus and planus, scoliosis, positive wrist and thumb sign, increased arm range/height ratio, reduced upper segment/lower segment ratio, striae distensae, easy bruising, translucent skin, strabismus divergens, high myopia (-4.5 diopters) and dural ectasia (lumbar) were diagnosed. A missense variant in exon 7 within *TGFBR2* (NM_003242.6: c.1582C>G (p.Arg528Gly)] was also detected by Sanger sequencing. Arginine was exchanged with glycine at position 528. ClinVar (http://www.ncbi.nlm.nih.gov/clinvar/) and the Human Gene Mutation Database (http://www.hgmd.cf.ac.uk/ac/index.php) do not cite this variant, but other missense alterations affecting arginine^528^ are listed as pathogenic variants. Unfortunately, genetics testing could not be performed on the clinically unaffected parents to prove the de novo status of this variant. The highly conserved kinase domain was also affected. The function of the receptor should be modified. The aortic diameters were assessed frequently. From 39 mm (*z*-score: +7.86) at the time of his first presentation, the diameter of his sinus of Valsalva increased to 46 mm (*z*-score: +9.5) over a period of 22 months [4]. Additionally, a persistent ductus arteriosus, sinus arrhythmia and an enlarged heart were seen. The aortic and mitral valves showed mild regurgitation (Table 1).

## FOLLOW-UP

According to the current recommendations, the 2 young patients in our series underwent valve-sparing aortic root replacement at the ages of 4 and 5 years, respectively. The 2 patients are free from reoperation, adverse neurological events or aortic regurgitation after 8 years (patient A) and 2 years (patient B), respectively.

## DISCUSSION

We present 2 patients with LDS, whose missense alterations in exon 7 of *TGFBR2* lie very close to each other, only 5 codons apart (p.Arg528Gly and p. Cys533Arg). Both alterations of the corresponding protein affect the highly conserved kinase domain. Unfortunately, no skin biopsy was taken from either affected individual, which made it impossible to further investigate changes of TGF-β signaling due to the observed variants in the cultivated fibroblasts. The genetic proximity, however, did not reflect the significant variation of aneurysm location in the ascending aorta. From an embryological point of view, the thoracic aorta is a heterogeneous structure whose segments develop one after the other with a specific set of signaling pathways and genes. The 2 presented cases depict the independence with which their contrary aneurysms are formed despite their similar genotypes. *TGFBR2* missense variants leading to LDS alter amino acids in the intracellular domain of the receptor and are predicted to disrupt kinase activity of the receptors and thus likely prevent proper signaling, thereby disrupting the differentiation of the neural crest and the mesenchymal cells into vascular smooth muscle cells (SMC). SMCs from LDS may instead increase TGF-β in an attempt to drive SMC differentiation or increase extracellular matrix deposition [5]. The effect of TGF-β on the smooth muscle cell gene expression differs depending on the location of the smooth muscle cells in the aorta. TGF-β isoforms have different effects on the smooth muscle transcriptional response in a lineage-dependent manner, with the highest response occurring in lineage of ectodermal origin (the outflow tract and proximal ascending aorta) versus the lowest response in the mesodermal lineage (dorsal aorta) [6]. Histopathological findings depict these alterations. LDS shows diffuse medial degeneration and increased collagen deposition (in contrast to Marfan syndrome) [7]. Abnormal TGF-β signaling by TGFBR mutations in different parts of the ascending aorta may result in increased diffuse medial degeneration and collagen deposition that are also specific for the corresponding region. The 2 presented cases may show the clinical correlate of this mechanism. The composition of the density of TGFBR mutations and therefore the increased TGF-β signaling, by an immunohistochemical stain for pSmad2, a marker of TGF*-*β activity in different parts of the ascending aorta, may be part of future investigations.

The literature contains a relative paucity of information regarding the identification of the genetic and molecular factors that influence the development and differentiation of the aortic root and the ascending aorta. However, a single gene, receptor or signaling pathway does not seem to have a generalized effect on the development of specific segments of the aortic tree. We agree with the authors of previous investigations that, for the precise identification of genotype-phenotype correlations, larger databases containing both genotype- and phenotype data are needed.

**Funding**

None.

**Conflict of interest**: none declared.
